# Manipulation and Instability: Exploring Machiavellianism and Borderline Personality Similarities and Differences

**DOI:** 10.3390/ejihpe15090185

**Published:** 2025-09-12

**Authors:** Bruno Bonfá-Araujo, Christian Blötner, András Láng, Julie Aitken Schermer

**Affiliations:** 1 Department of Psychology, The University of Western Ontario, London, ON N6A 3K7, Canada; 2 Department of Forensic Psychology, Universidade Tuiuti do Paraná, Curitiba 82010-210, Brazil; 3 Department of Psychology, FernUniversität in Hagen, 58097 Hagen, Germany; christian.bloetner@fernuni-hagen.de; 4 Institute of Psychology, University of Pécs, 7624 Pécs, Hungary; lang.andras@pte.hu; 5 Departments of Psychology and Management and Organizational Studies, The University of Western Ontario, London, ON N6A 3K7, Canada; jharris@uwo.ca

**Keywords:** emotional regulation, emotion dysfunction, Big Five, Latent Profile Analysis

## Abstract

Machiavellianism and borderline personality are known for influencing interpersonal dynamics through manipulative behaviors. Machiavellianism is characterized by calculated, egotistic, and callous manipulation, while borderline personality involves emotionally driven impulsive manipulation due to instability and fear of abandonment. In this study, we explored the relationships of the two constructs with respect to broader personality constructs. Adult participants (N = 1011; Mage = 49.08 years, SD = 17.15) completed two measures each for Machiavellianism and borderline personality and a single inventory measuring the Big Five personality traits. Latent Profile Analysis (LPA) was used to investigate subgroups within the data. Machiavellianism was more strongly negatively associated with agreeableness and conscientiousness, while borderline personality traits were more strongly linked to neuroticism (more positively), agreeableness, and conscientiousness (both more negatively). Two distinct latent profiles emerged. Based on these findings, we suggest that Machiavellianism can align with either adaptive or maladaptive functioning, whereas a combination of Machiavellianism and borderline personality traits underscores a tendency towards manipulative behaviors with emotional instability. We suggest that future research build upon our findings by investigating concrete manipulative acts predicted by borderline personality and Machiavellianism.

## 1. Introduction

Interpersonal manipulation is a behavior that can damage trust and cooperation in both personal and organizational settings, yet the factors that drive this behavior are not fully understood (Williams & Muir, 2019). The present study aims to address this gap by exploring how two conceptually different constructs, Machiavellianism and borderline personality, show distinct patterns of manipulation. Specifically, we intend to clarify the unique links each trait has with the Five-Factor Model of personality and identify latent profiles that reveal manipulation patterns. This research sets the stage for further studies into specific manipulative behaviors, from cold, instrumental tactics to emotionally charged control efforts.

Machiavellianism, named after the philosopher Niccolò Machiavelli, is characterized by the endorsement of manipulation, exploitation, and egotism (Christie & Geis, 1970; Rauthmann & Will, 2011). People with higher Machiavellianism scores often lack moral restraint in both interpersonal and organizational settings, and their actions are primarily driven by a deliberate tactic to maximize personal gains regardless of ethical concerns (Aldousari & Ickes, 2021). This being said, Machiavellianism is not the only trait that is associated with interpersonal manipulation. Given distinct motives and circumstances driving concrete behaviors, it is important to identify which individual difference variable in particular drives which act of manipulation. For instance, *borderline personality disorder* (BPD) is characterized by emotional instability, intense and unstable interpersonal relationships, and a persistent fear of abandonment (American Psychiatric Association, 2022). BPD involves attempts to control or influence others through emotional outbursts, threats of self-harm, or rapidly shifting expressions of affection as a way to stave off perceived abandonment. Furthermore, people with higher scores on measures of borderline personality exhibit behaviors such as mood swings, impulsive actions, and an unstable self-image (Alfieri et al., 2025; Leichsenring et al., 2023). Considering these characterizations, it stands to reason that Machiavellianism and borderline personality manifest differently with respect to manipulative acts. This study provides initial evidence for differential expressions of Machiavellianism and borderline personality with respect to broad personality traits to enable subsequent tests of the relations of Machiavellianism and borderline personality with specific manipulative acts concomitant with specific personality markers (e.g., emotionally driven manipulation versus strategic manipulation).

### 1.1. Commonalities and Differences Between Machiavellianism and Borderline Personality

Considering that both Machiavellianism and borderline personality are associated with socially undesirable inclinations, certain overlaps are possible (Láng, 2015; Nasab et al., 2023); however, both traits have distinct associations within broad personality models, such as the *Five-Factor Model* (i.e., openness, conscientiousness, extraversion, agreeableness, and neuroticism). People with high scores in Machiavellian traits tend to score low in agreeableness and high in conscientiousness because of their manipulative tendencies and lack of regard for conventional morality, whereas the role of neuroticism is under debate (Blötner et al., 2025; Collison et al., 2018). For instance, Collison et al. (2018) postulated emotional invulnerability as a component of Machiavellianism, whereas Monaghan et al. (2016) advocate for the role of elevated neuroticism in explaining Machiavellianism. In contrast, borderline personality is strongly associated with intense emotional instability and anxiety (i.e., high neuroticism; Streit et al., 2022). People who score higher on measures of borderline personality also tend to score lower on agreeableness, but for different reasons compared to Machiavellianism; behaviors related to borderline personality are driven by emotional volatility and impulsivity rather than calculated, self-enhancing manipulation (Collison et al., 2018; Distel et al., 2009). The different but somewhat similar patterns of association between Machiavellianism and borderline personality within the Five-Factor Model underscore the differences in their underlying structures and provide insights into how these traits manifest in interpersonal relationships.

The similarities and divergences of Machiavellianism and borderline personality offers a lens through which to examine manipulative behaviors. Machiavellianism and borderline personality involve manipulation, but the nature and motivations underlying these behaviors are clearly singular (Láng, 2015). Machiavellianism involves callous, calculated manipulation with little concern for others, primarily aimed at achieving predetermined self-serving goals when the benefits outweigh the costs (Jones & Mueller, 2022; Rauthmann & Will, 2011). In contrast, the manipulations of people with borderline personality disorder are impulsive and driven by unstable emotional states and a need for emotional closeness or stability (Links et al., 1999). Contrasting the calculated manipulative tactics of Machiavellianism, people with high levels of borderline personality traits enact manipulative behaviors driven by acute emotional distress and an urgent need for security and acceptance. These behaviors are not strategic, but are reactions to the fear of abandonment and identity instability (Links et al., 1999).

Machiavellianism is a key component of the *Dark Triad*, a constellation of aversive personality traits known to be detrimental to interpersonal relationships (Paulhus & Williams, 2002). Borderline personality, in its subclinical version, is discussed as part of the *Vulnerable Dark Triad*, expanding the traditional Dark Triad to include traits characterized by emotional instability (Miller et al., 2010). This expanded triad incorporates elements such as hypersensitivity and vulnerability (Bonfá-Araujo & Schermer, 2024). It highlights the intersection of manipulative behaviors with emotional vulnerability and distinguishes impulsive, fear-driven manipulation (which is typical of borderline personality disorder) from the calculated strategies characteristic of Machiavellianism. Our study expands this research by empirically analyzing specific subdimensions related to broader personality traits, especially agreeableness. Clinically, this distinction matters because negative tactics and antagonism are linked to maladaptive, antisocial tendencies (Paulhus & Williams, 2002), whereas positive tactics and planfulness may support seemingly adaptive social functioning. Therefore, this study offers a new perspective for the VDT literature and highlights the clinical importance of examining these subdimensions in real-world settings.

### 1.2. Current Study

This research aims to further understand the relationship between Machiavellianism and borderline personality. We seek to explore the similarities and differences between these traits and their associations within a broad personality model. By comparing Machiavellianism and borderline personality, we can gain better insights into the mechanisms of manipulation and control and how they are differently associated with broad personality traits. Based on the literature, we hypothesize that Machiavellianism will be positively associated with borderline personality (H1; Láng, 2015) and negatively associated with agreeableness (H2; Collison et al., 2018). Borderline personality is predicted to be positively associated with neuroticism (H3) and negatively associated with agreeableness (H4) and conscientiousness (H5; Collison et al., 2018; Distel et al., 2009; Streit et al., 2022). Finally, we expect to find two separable profiles in Latent Profile Analysis, thus differentiating Machiavellianism and borderline personality traits (H6), both of which yield different associations with Big Five traits.

## 2. Method

### 2.1. Participants and Procedures

We surveyed 1011 financially compensated panelists of the Leger community to participate in our study; 50.44% identified as female, 49.35% identified as male, and 0.09% preferred not to say, with a mean age of 49.08 years (*SD* = 17.15, age range 18–89). After providing informed consent, participants completed the survey through Qualtrics. The study received ethical approval (protocol 2023-123719-84408) from the university’s non-medical research ethics board. The database can be accessed at https://osf.io/d3f96/?view_only=5c3e0a09feec4f328c2138cd1ba81f86 (accessed on 8 September 2025).

### 2.2. Measures

To assess Machiavellianism, we used two measures: the 20-item Mach IV scale (Christie & Geis, 1970), answered using a Likert-type scale (1 = *Strongly disagree* to 7 = *Strongly agree*), and the 52-item *Five Factor Machiavellianism Inventory* (FFMI; Collison et al., 2018), answer using a Likert scale (1 = *Disagree strongly* to 5 = *Agree strongly*). The Mach IV scale comprises the facets *Negative Interpersonal Tactics*, *Positive Interpersonal Tactics*, *Cynical View Human Nature*, and *Positive View Human Nature*, as proposed by Corral and Calvete (2000). The FFMI has 13 dimensions (i.e., Achievement, Activity, Selfishness, Assertiveness, Callousness, Competence, Cynical, Deliberation, Invulnerable, Immodesty, Order, Self-Confidence, and Manipulative), which can be summed into three factors, Agency, Antagonism, and Planfulness. In addition, Collison et al. (2018) recommended computation of an overall score of the FFMI. To assess borderline personality, we used two measures: the 31-item *Personality Assessment Inventory—Borderline Features* (PAI-BOR; Morey, 1991), answered using a Likert-type scale (1 = *False, not at all true* to 4 = *Very true*), and the 48-item *Five Factor Borderline Personality Inventory—Short Form* (FFBI-SF; DeShong et al., 2016), answered using a Likert scale (1 = *Disagree strongly* to 5 = *Agree strongly*). The PAI-BOR has four dimensions (i.e., Affective Instability, Identity Problems, Negative Relationships, and Self-Harm), while the FFBI has 12 dimensions (i.e., Affective Dysregulation, Anxious Uncertainty, Behavioral Dysregulation, Despondence, Dissociative Tendencies, Distrustfulness, Dysregulated Anger, Fragility, Manipulativeness, Oppositional, Rashness, and Self-Disturbance). Given the high level of abstraction when using the 12 dimensions of the FFBI, we decided to compute an overall score. We selected two measures for each construct to enhance both theoretical alignment and methodological clarity. First, by pairing the FFMI and FFBI scales, grounded directly in the Five-Factor Model, we can more precisely evaluate how Machiavellianism and borderline features map onto established personality domains. Second, including the classic MACH-IV and PAI-BOR alongside these newer, model-based instruments helps to disentangle whether observed differences in manipulative tendencies arise from the constructs themselves or from idiosyncrasies of particular measures. Reliability estimates for all variables (McDonald’s ωs) can be found in Table 1.

The Big Five personality traits were assessed using the 44-item *Big Five Inventory* (BFI; John & Srivastava, 1999), answered using a Likert scale (1 = *Disagree strongly* to 5 = *Agree strongly*). The BFI has five dimensions (i.e., Agreeableness, Conscientiousness, Extraversion, Neuroticism, and Openness). Reliability estimates for all variables (McDonald’s ωs) can be found in Table 1.

### 2.3. Data Analysis

First, we tested bivariate Pearson’s correlations considering the factors within each scale. Given the relatively large sample size, statistical power suffices even for negligible correlation coefficients to yield significance. In line with this, correlations as high as |*r*| ≈ 0.10 yield significance at α = 0.05, given *n* = 1011, 1 − β = 0.95, one-sided test (Champely et al., 2020). Thus, we treated correlations as low if <0.20, as high if >0.50, and as moderate if they were in between. Next, we performed a *Latent Profile Analysis* (LPA), considering the facets within each scale, which allowed the investigation of subgroups within a population (Muthén, 1989). LPA was selected because it enables the identification of distinct subgroups within a heterogeneous population based on participants’ responses (Muthén, 1989; Nylund-Gibson & Choi, 2018). It allowed us to uncover qualitatively distinct groups characterized by unique constellations of Machiavellianism and borderline personality traits. Additionally, by comparing the Big Five personality trait distributions across these latent profiles, we aimed to provide deeper insights into how distinct manipulative tendencies show different associations with broader personality traits. We used the recommendations made by Nylund et al. (2007) to retain the best profiles; that is, Akaike Information Criterion (AIC), Bayesian Information Criterion (BIC), and Sample-Adjusted BIC (SBIC) with lower values indicating better fit. The Bootstrapped Likelihood Ratio Test (BLRT) and entropy (with values above 0.80 being desirable) were also considered. All analyses were performed in RStudio (Version 2025.05.1+513, R Core Team, 2020) using the packages *tidyLPA* (Rosenberg et al., 2018), *dplyr* (Wickham et al., 2023), *ggplot2* (Wickham, 2016), *rstatix* (Kassambara, 2023), and *tidyr* (Wickham & Girlich, 2023).

## 3. Results

Table 1 reports the correlations among the Big Five, Machiavellianism, and Borderline Personality. The results show that the negative and cynical aspects of Machiavellianism, as measured by the MACH-IV, are strongly positively associated with the antagonistic traits as measured by the FFMI, while the positive traits from the former aspects are negatively associated with the latter or only at negligible levels (i.e., *r*s < 0.20). Borderline traits, as measured by the FFBI and the PAI-BOR, are positively and strongly correlated with each other. Moreover, the positive and aversive facets of Machiavellianism (i.e., positive tactics, positive views, agency, and planfulness vis-à-vis negative tactics, cynical views, and antagonism) yield moderate yet inverse associations with borderline traits.

On the one hand, the aversive aspects of Machiavellianism (i.e., negative tactics, cynical views, and antagonism) are negatively associated with extraversion, neuroticism, and especially with agreeableness and conscientiousness, while the more positive aspects (i.e., positive tactics, positive views, agency, and planfulness) are positively related to extraversion, agreeableness, and conscientiousness and negatively related to neuroticism. On the other hand, borderline traits are negatively associated with extraversion, agreeableness, conscientiousness, and openness, while they are highly and positively correlated with neuroticism. We provide a more detailed correlation table with all first-order facets of the FFMI and the FFBI in the Supplementary Materials.

Latent Profile Analyses using MACH-IV, FFMI, PAI-BOR and FFBI factors were tested, allowing for up to six profiles. The results are shown in Table 2. All solutions showed that entropy levels above the desired level of 0.80. AIC, BIC, and SABIC indicators decreased with every possible solution, which is not uncommon and should be considered carefully because it does not always mean the model with higher classes is the most suitable (Nylund-Gibson & Choi, 2018). Based on an elbow plot (Figure S1), we opted for the two-class model because the largest declines in the trajectories of the AIC and BIC occurred in the transition from the one-class model to the two-class model. Subsequent changes (e.g., from the two-class model to the three-class model) were comparatively small (Sinha et al., 2021).

**Figure 1:**
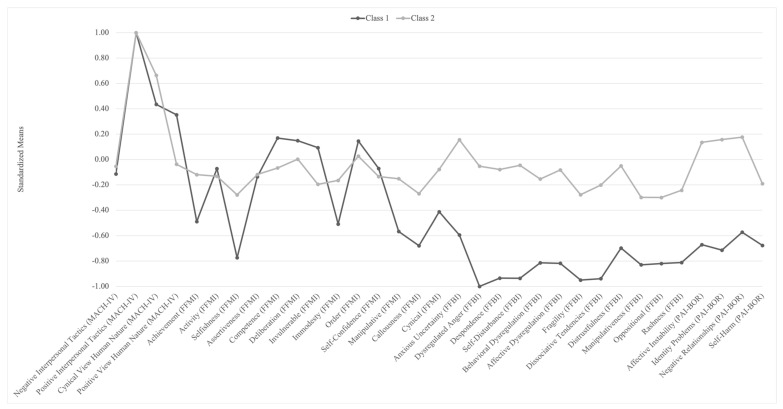
Standardized means for profiles scores.

Figure 1 plots the profiles with standardized means. Class 1 (compared to Class 2) showed higher scores in positive aspects of Machiavellianism, such as Competence (FFMI), Deliberation (FFMI), and Invulnerable (FFMI). Class 2 (compared to Class 1) showed higher scores for the Negative View of Human Nature (MACH-IV) and all aspects from both borderline measures (i.e., FFBI and PAI-BOR). The two classes showed similar scores for both Positive and Negative Interpersonal Tactics.

We conducted independent samples t-tests comparing Class 1 (*n* = 316) and Class 2 (*n* = 510) for all Machiavellianism and borderline personality facets. Class 2 (borderline-dominant) scored significantly higher than Class 1 (Machiavellian-dominant) on Negative Interpersonal Tactics (t = −8.63, *p* < 0.001, d = −0.63), Cynical View of Human Nature (t = −8.69, *p* < 0.001, d = −0.62), Manipulative (t = −8.27, *p* < 0.001, d = −0.59), and Callousness (t = −6.72, *p* < 0.001, d = −0.46), as well as Selfishness, Cynical, and Immodesty (all *p* < 0.001, d = −0.30 to −0.46). Conversely, Class 1 showed significantly higher means and medium-to-large effect sizes on agentic and planful Machiavellian facets, such as Activity (t = 12.07, *p* < 0.001, d = 0.87), Assertiveness (t = 4.18, *p* < 0.001, d = 0.30), Competence (t = 15.65, *p* < 0.001, d = 1.12), Deliberation (t = 7.93, *p* < 0.001, d = 0.55), Invulnerable (t = 20.17, *p* < 0.001, d = 1.45), Order (t = 6.10, *p* < 0.001, d = 0.44), and Self-Confidence (t = 11.29, *p* < 0.001, d = 0.80). For Positive Interpersonal Tactics, Class 1 also scored higher (t = 7.55, *p* < 0.001, d = 0.54). Class 2 had markedly higher scores on all borderline and dysregulation variables, including Anxious Uncertainty (t = −19.47, *p* < 0.001, d = −1.46), Dysregulated Anger (t = −23.53, *p* < 0.001, d = −1.77), Despondence (t = −21.06, *p* < 0.001, d = −1.61), Self-Disturbance (t = −26.38, *p* < 0.001, d = −1.99), Behavioral Dysregulation (t = −24.06, *p* < 0.001, d = −1.80), Affective Dysregulation (t = −26.26, *p* < 0.001, d = −1.94), Fragility, Dissociative Tendencies, Distrustfulness, Manipulativeness, Oppositional, and Rashness (all *p* < 0.001, d = −0.80 to −1.30). The same held for Affective Instability (t = −26.36, *p* < 0.001, d = −2.02), Identity Problems (t = −24.00, *p* < 0.001, d = −1.83), Negative Relationships (t = −21.70, *p* < 0.001, d = −1.56), and Self-Harm (t = −13.04, *p* < 0.001, d = −0.96).

Mean comparison tests indicated that Class 2 scored significantly higher than Class 1 on key Machiavellianism dimensions related to antagonism and manipulativeness (e.g., Negative Interpersonal Tactics, Manipulative, and Callousness), with moderate effect sizes (d ≈ 0.50–0.70). In contrast, Class 1 showed notably higher means and large effect sizes on dimensions related to agentic, strategic, and planful manipulation (e.g., Activity, Competence, Invulnerable, and Self-Confidence; d = 0.80–1.45). Both groups differed robustly across all borderline and dysregulation facets, with Class 2 exhibiting much higher scores.

## 4. Discussion

The aim of this research was to further understand the relationship between Machiavellianism and borderline personality. We found that Machiavellianism was positively associated with extraversion and conscientiousness and negatively associated with agreeableness and neuroticism. Although previous results only converge on Machiavellianism’s negative association with agreeableness (Paulhus & Williams, 2002), other associations could be well understood if we identify these traits as part of a Machiavellian exploitative strategy (Jones & Figueredo, 2013). Being extraverted could increase social reputation and social embeddedness (Ashton et al., 2000) and grant a person the charm necessary for manipulation (O’Boyle et al., 2012), and more conscientious individuals are good at planning ahead (Jones & Paulhus, 2009). Being rather introverted, in turn, would reflect Machiavellian mistrust and misanthropy and could reduce the likelihood of being exploited by others (Christie & Geis, 1970; Monaghan et al., 2016, 2018). Along with higher emotional stability, these could be elements of the strategic nature of Machiavellianism (Blötner et al., 2025; Fehr et al., 1992; Jonason & Webster, 2012), which has already been described by Christie and Geis (1970) as the “cool syndrome”.

Borderline personality traits were positively associated with neuroticism and negatively associated with extraversion, agreeableness, and conscientiousness, aligning with the substantial evidence depicting borderline personality as a maladaptive variant of the Five-Factor model of personality (Saulsman & Page, 2004; Trull et al., 2003). Regarding the association of Machiavellianism and borderline personality, antagonistic characteristics of Machiavellianism were positively associated with borderline personality traits, which we attribute (at least in part) to commonalities with respect to resorting to certain manipulative interpersonal means to fulfill one’s needs (e.g., Christie & Geis, 1970; Leichsenring et al., 2023; Nasab et al., 2023). Components of borderline personality, such as identity diffusion or affective instability, could contribute—at least at a subclinical level—to being flexible and versatile, which in turn could increase one’s success at manipulating others (Láng, 2015). Further, antagonism could also be a common feature of borderline personality and Machiavellianism (Fossati et al., 2007; Miller et al., 2010; Paulhus & Williams, 2002).

Based on Latent Profile Analysis, we discriminated between two classes using responses to Machiavellianism and borderline personality measures. While scoring similarly on both Positive and Negative Interpersonal Tactics (MACH-IV), scores of views about human nature and borderline personality traits (as measured by both FFBI and PAI-BOR) differed for the two classes. Interpreting these profiles using personality theory, we suggest that one profile exhibits a more “strategic–manipulative” setup (marked by high planfulness and agency). In contrast, the other shows a “reactive–dysregulated” setup (characterized by high affective instability and antagonism). This difference aligns with the adaptive–maladaptive spectrum in personality psychology and fits with clinical models that distinguish between instrumental manipulation and emotionally driven interpersonal problems. These results highlight that Machiavellian interpersonal tactics could be combined with either adaptive or maladaptive personality functioning. Thus, we can refine Christie and Geis’ (1970) statement about Machiavellianism’s lack of association with gross psychopathology. Machiavellianism can (though that does not necessarily mean that it should) be associated with borderline personality traits that could be treated as a general factor of personality pathology (Sharp et al., 2015). Overall, our means-level analysis complicates the conventional view that strategic manipulation is unique to Machiavellianism, as many antagonistic and manipulative behaviors are equally, if not more, prominent among those high in borderline features. The clearest point of differentiation lies in the strategic, planful, and emotionally detached nature of manipulation in Class 1, as opposed to the emotionally volatile and dysregulated manipulation observed in Class 2. This pattern suggests that “strategic” manipulation should be conceptualized as a distinct, narrower facet within Machiavellianism, whereas broader manipulative behaviors may represent a marker of personality pathology.

A key limitation of this study is its reliance on self-report measures, which can be prone to social desirability biases. Furthermore, while the sample size is robust, the cross-sectional design limits causal inferences about the relationships between the constructs. Concerning sample diversity, it predominantly represents individuals from a specific cultural and geographic context (i.e., North America), which may limit the generalizability of our findings. Prior research (e.g., Blötner et al., 2025) has underscored the need to expand personality assessment to more diverse and underrepresented populations, as both Machiavellianism and Borderline traits may manifest differently across cultures, age groups, and sociocultural environments (Blötner et al., 2025; Munson et al., 2022). We encourage future research to address these gaps by replicating the current study in more heterogeneous samples and examining culturally specific expressions and implications of these personality constructs.

In conclusion, our study highlights the complex interplay between Machiavellianism and borderline personality traits, emphasizing their shared but distinct pathways to manipulative behaviors. Our findings gain particular significance when viewed at the intersection of personality and clinical psychology, reflecting a growing consensus that dimensional models offer richer explanatory power than categorical diagnoses alone. By identifying profiles, such as distinguishing strategic–manipulative from reactive–dysregulated tendencies, we contribute to ongoing efforts to integrate personality trait frameworks with clinical assessment practices. While Machiavellianism is driven by strategic and callous calculations, borderline personality manipulations are rooted in emotional instability and a desire for security. These findings contribute to a broader understanding of personality and provide a nuanced perspective on how manipulative behaviors emerge in both adaptive and maladaptive contexts.

## Figures and Tables

**Table 1:** Correlations between Machiavellianism, borderline personality, and the Big Five.

	ω	1	2	3	4	5	6	7	8	9	10	11	12	13	14	15	16	17
1. Negative Interpersonal Tactics (MACH-IV)	0.60																	
2. Positive Interpersonal Tactics (MACH-IV)	0.71	**−0.11**																
3. Cynical View of Human Nature (MACH-IV)	0.64	**0.59**	0.01															
4. Positive View of Human Nature (MACH-IV)	0.46	−0.08	**0.30**	−0.06														
5. Antagonism (FFMI)	0.75	**0.58**	**−0.37**	**0.52**	**−0.29**													
6. Agency (FFMI)	0.80	**−0.13**	**0.17**	**−0.12**	**0.11**	−0.05												
7. Planfulness (FFMI)	0.67	**−0.14**	**0.16**	**−0.18**	0.04	**−0.24**	**0.20**											
8. FFMI Total	0.73	**0.19**	−0.03	**0.15**	−0.07	**0.47**	**0.81**	**0.32**										
9. FFBI Total	0.95	**0.35**	**−0.24**	**0.32**	**−0.19**	**0.41**	**−0.52**	**−0.37**	**−0.28**									
10. Affective Instability (PAI-BOR)	0.81	**0.27**	**−0.19**	**0.24**	**−0.17**	**0.31**	**−0.50**	**−0.26**	**−0.28**	**0.83**								
11. Identity Problems (PAI-BOR)	0.79	**0.25**	**−0.15**	**0.21**	**−0.14**	**0.24**	**−0.48**	**−0.23**	**−0.30**	**0.78**	**0.72**							
12. Negative Relationships (PAI-BOR)	0.72	**0.29**	**−0.16**	**0.28**	**−0.22**	**0.33**	**−0.37**	**−0.25**	**−0.17**	**0.72**	**0.66**	**0.70**						
13. Self-Harm (PAI-BOR)	0.73	**0.22**	**−0.13**	**0.25**	−0.09	**0.30**	**−0.27**	**−0.53**	**−0.19**	**0.61**	**0.52**	**0.49**	**0.42**					
14. Extraversion	0.84	**−0.15**	**0.10**	−0.04	**0.12**	**−0.12**	**0.62**	−0.07	**0.38**	**−0.25**	**−0.29**	**−0.26**	**−0.23**	−0.03				
15. Agreeableness	0.81	**−0.36**	**0.37**	**−0.32**	**0.29**	**−0.67**	**0.31**	**0.26**	−0.07	**−0.55**	**−0.48**	**−0.33**	**−0.43**	**−0.35**	**0.28**			
16. Conscientiousness	0.80	**−0.23**	**0.27**	**−0.20**	**0.15**	**−0.29**	**0.60**	**0.58**	**0.47**	**−0.57**	**−0.48**	**−0.45**	**−0.37**	**−0.53**	**0.30**	**0.45**		
17. Neuroticism	0.88	**0.17**	**−0.21**	**0.14**	**−0.17**	**0.17**	**−0.62**	**−0.15**	**−0.42**	**0.75**	**0.74**	**0.68**	**0.59**	**0.34**	**−0.36**	**−0.41**	**−0.47**	
18. Openness	0.79	−0.08	0.09	**−0.16**	0.06	**−0.10**	**0.38**	0.07	**0.26**	**−0.11**	**−0.12**	−0.09	−0.03	−0.07	**0.33**	**0.24**	**0.24**	**−0.18**

*Note.* Bold correlations are *p* < 0.001; ω = McDonald’s omega.

**Table 2:** Model Fit Index for the Latent Profile Analysis.

Classes	LL	AIC	BIC	SABIC	BLRT_p	Entropy	Prob. min	Prob. max	n min	n max
1	−54,868	109,867.92	110,159.64	109,950		1	1	1	1	1
2	−51,919	104,038.55	104,480.54	104,163	0.01	0.96	0.98	0.99	0.37	0.63
3	−51,132	102,532.4	103,124.68	102,699	0.01	0.94	0.96	0.98	0.22	0.44
4	−50,739	101,814.42	102,556.98	102,024	0.01	0.95	0.93	0.99	0.18	0.42
5	−50,297	100,998.15	101,890.99	101,250	0.01	0.94	0.94	0.98	0.13	0.31
6	−50,054	100,580.53	101,623.65	100,874	0.01	0.93	0.92	0.99	0.13	0.22

*Notes.* LL—log likelihood; AIC—Akaike Information Criteria; BIC—Bayesian Information Criterion; SABIC—samples-adjusted BIC; BLRT—bootstrap likelihood ratio test; Prob. min—minimum probability of profile membership; Prob. max—maximum probability of profile membership.

## Data Availability

The original data presented in the study are openly available at [https://osf.io/d3f96/?view_only=5c3e0a09feec4f328c2138cd1ba81f86].

## Supplementary Materials

The following supporting information can be downloaded at: https://www.mdpi.com/article/10.3390/ejihpe15090185/s1, Figure S1: Elbow plot; Table S1: Sex differences for Machiavellianism and Borderline Personality; Table S2: Correlations between Machiavellianism, Borderline Personality, and the Big Five.
