# Supplementary material for: Manipulation and Instability: Exploring Machiavellianism and Borderline Personality Similarities and Differences
Source: Eur J Investig Health Psychol Educ. 2025 Sep 12;15(9):185. doi: 10.3390/ejihpe15090185 (PMC12469004; doi:10.3390/ejihpe15090185)

Manipulation and Instability: Exploring Machiavellianism and Borderline Personality Similarities and Differences

We initially tested the mean comparisons between men and women. The results are shown in Table S1. Men showed higher means for Negative Interpersonal Tactics, Selfishness, Invulnerable, Immodesty, Self-Confidence, Manipulative, Callousness, and Manipulativeness. Women showed higher means for Anxious Uncertainty, Despondence, Affective Dysregulation, Identity Problems, Negative Relationships, and Neuroticism. In summary, men showed higher scores for antagonistic traits, while women showed higher scores for emotionally unstable behaviors.

Table S1.

Sex differences for Machiavellianism and Borderline Personality.

	t	*df*	*p*	*d*	M_{Men}	SD_{Men}	M_{Women}	SD_{Women}
**Negative Interpersonal Tactics (MACH-IV)**	**-3.792**	**1003**	**< .001**	**-0.239**	**15.05**	**4.67**	**13.97**	**4.37**
Positive Interpersonal Tactics (MACH-IV)	-0.966	1000	0.334	-0.061	31.20	5.84	30.85	5.81
Cynical View Human Nature (MACH-IV)	-2.898	999	0.004	-0.183	20.92	6.03	19.86	5.53
Positive View Human Nature (MACH-IV)	-1.500	999	0.134	-0.095	17.42	3.77	17.06	3.81
Achievement (FFMI)	0.604	1000	0.546	0.038	11.34	3.79	11.48	3.68
Activity (FFMI)	-0.513	999	0.608	-0.032	13.55	3.24	13.45	3.23
**Selfishness (FFMI)**	**-5.527**	**999**	**< .001**	**-0.349**	**7.81**	**2.74**	**6.91**	**2.41**
Assertiveness (FFMI)	-2.892	1005	0.004	-0.182	13.17	3.21	12.56	3.37
Competence (FFMI)	-0.995	997	0.32	-0.063	14.88	3.13	14.68	3.20
Deliberation (FFMI)	-0.414	998	0.679	-0.026	14.93	3.21	14.85	3.16
**Invulnerable (FFMI)**	**-8.531**	**1003**	**< .001**	**-0.538**	**14.43**	**3.49**	**12.52**	**3.61**
**Immodesty (FFMI)**	**-6.438**	**1005**	**< .001**	**-0.406**	**11.49**	**2.82**	**10.32**	**2.92**
Order (FFMI)	0.889	1001	0.374	0.056	15.04	2.95	15.21	3.15

**Self-Confidence (FFMI)**	**-6.123**	**997**	**< .001**	**-0.387**	**14.04**	**3.12**	**12.75**	**3.51**
**Manipulative (FFMI)**	**-4.055**	**1004**	**< .001**	**-0.256**	**10.88**	**3.24**	**10.06**	**3.21**
**Callousness (FFMI)**	**-8.491**	**1003**	**< .001**	**-0.536**	**9.59**	**3.38**	**7.88**	**3.00**
Cynical (FFMI)	-0.126	997	0.899	-0.008	11.70	2.97	11.68	3.06
**Anxious Uncertainty (FFBI)**	**8.371**	**1001**	**< .001**	**0.529**	**10.37**	**4.40**	**12.74**	**4.55**
Dysregulated Anger (FFBI)	-0.481	995	0.631	-0.030	8.67	4.27	8.54	4.48
**Despondence (FFBI)**	**5.190**	**1002**	**< .001**	**0.328**	**8.35**	**4.13**	**9.77**	**4.54**
Self-Disturbance (FFBI)	2.288	1001	0.022	0.144	9.02	4.06	9.64	4.50
Behavioral Dysregulation (FFBI)	-1.832	1004	0.067	-0.116	8.25	3.93	7.80	3.99
**Affective Dysregulation (FFBI)**	**3.720**	**1004**	**< .001**	**0.235**	**8.01**	**4.11**	**9.03**	**4.56**
Fragility (FFBI)	2.221	999	0.027	0.140	6.52	3.38	7.01	3.65
Dissociative Tendencies (FFBI)	1.903	1002	0.057	0.120	6.99	3.91	7.48	4.31
Distrustfulness (FFBI)	2.209	1003	0.027	0.139	9.65	4.16	10.25	4.46
**Manipulativeness (FFBI)**	**-5.649**	**1004**	**< .001**	**-0.356**	**7.62**	**3.59**	**6.42**	**3.12**
Oppositional (FFBI)	-2.937	1005	0.003	-0.185	7.38	3.19	6.81	2.95
Rashness (FFBI)	-2.520	1001	0.012	-0.159	7.72	3.76	7.13	3.62
Affective Instability (PAI-BOR)	2.629	993	0.009	0.167	10.76	3.52	11.39	4.00
**Identity Problems (PAI-BOR)**	**4.768**	**998**	**< .001**	**0.302**	**10.94**	**3.59**	**12.13**	**4.27**
**Negative Relationships (PAI-BOR)**	**4.112**	**996**	**< .001**	**0.260**	**11.98**	**3.49**	**12.94**	**3.84**
Self-Harm (PAI-BOR)	-0.268	1000	0.789	-0.017	9.27	2.97	9.22	3.16
Extraversion	1.851	994	0.064	0.117	23.25	6.44	24.02	6.62
Agreeableness	2.819	989	0.005	0.179	34.08	6.00	35.14	5.80
Conscientiousness	0.403	993	0.687	0.026	34.83	6.00	34.99	6.10
**Neuroticism**	**7.052**	**994**	**< .001**	**0.447**	**20.55**	**7.04**	**23.82**	**7.54**
Openness	-2.071	996	0.039	-0.131	34.86	6.30	34.02	6.55

Notes. Bold correlations are $p < .001$, t – Student's t, d – Cohen's d, M – Mean, SD – Standard Deviation, MACH-IV – Machiavellianism Scale – IV, FFMI – Five Factor Machiavellianism Inventory, FFBI – Five Factor Borderline Personality Inventory, PAI-BOR - Personality Assessment Inventory – Borderline Features.

Table S2 reports the correlations between the Five Factor Model and the facets of Machiavellianism and Borderline Personality. The results suggest that Machiavellianism, as measured by the MACH-IV and FFMI, is positively associated with extraversion and conscientiousness and negatively associated with agreeableness and neuroticism. Borderline Personality, as measured by the PAI-BOR and FFBI, is positively associated with neuroticism and negatively associated with extraversion, agreeableness, and conscientiousness. In addition, the antagonistic characteristics of Machiavellianism are positively associated with Borderline Personality.

Table S2.

Correlations between Machiavellianism, Borderline Personality, and the Big Five

	1	2	3	4	5	6	7	8	9	10	11	12	13	14	15	16
1. Extraversion	—															
2. Agreeableness	**0.28**	—														
3. Conscientiousness	**0.29**	**0.45**	—													
4. Neuroticism	**-0.36**	**-0.41**	**-0.48**	—												
5. Openness	**0.33**	**0.24**	**0.24**	**-0.18**	—											
6. Negative Interpersonal Tactics (MACH-IV)	**-0.15**	**-0.36**	**-0.23**	**0.17**	-0.08	—										
7. Positive Interpersonal Tactics (MACH-IV)	**0.10**	**0.37**	**0.27**	**-0.21**	0.09	**-0.11**	—									
8. Cynical View Human Nature (MACH-IV)	-0.04	**-0.32**	**-0.20**	**0.14**	**-0.16**	**0.59**	0.01	—								
9. Positive View Human Nature (MACH-IV)	**0.12**	**0.29**	**0.15**	**-0.17**	0.06	-0.08	**0.30**	-0.06	—							
10. Achievement (FFMI)	**0.29**	-0.03	0.09	0.01	**0.21**	**0.23**	-0.03	**0.18**	-0.03	—						
11. Activity (FFMI)	**0.46**	**0.32**	**0.56**	**-0.40**	**0.24**	**-0.12**	**0.18**	-0.08	**0.11**	**0.36**	—					
12. Selfishness (FFMI)	**-0.21**	**-0.57**	**-0.38**	**0.16**	**-0.30**	**0.38**	**-0.38**	**0.37**	**-0.21**	0.06	**-0.23**	—				
13. Assertiveness (FFMI)	**0.61**	0.09	**0.34**	**-0.32**	**0.36**	**-0.13**	0.05	**-0.11**	0.02	**0.23**	**0.35**	**-0.16**	—			
14. Competence (FFMI)	**0.44**	**0.34**	**0.64**	**-0.52**	**0.28**	**-0.22**	**0.18**	**-0.22**	**0.15**	**0.18**	**0.58**	**-0.30**	**0.50**	—		

15. Deliberation (FFMI)	**-0.14**	**0.23**	**0.42**	**-0.11**	0.09	**-0.15**	**0.14**	**-0.22**	0.03	**-0.11**	**0.11**	**-0.23**	-0.02	**0.17**	—	
16. Invulnerable (FFMI)	**0.29**	**0.31**	**0.50**	**-0.78**	**0.21**	**-0.21**	**0.16**	**-0.20**	**0.12**	-0.01	**0.40**	**-0.17**	**0.36**	**0.55**	**0.23**	—
17. Immodesty (FFMI)	**0.13**	**-0.26**	-0.02	-0.05	**0.24**	**0.37**	**-0.13**	**0.31**	-0.06	**0.36**	0.10	**0.29**	**0.22**	0.09	**-0.12**	0.06
18. Order (FFMI)	0.02	**0.22**	**0.58**	**-0.15**	0.03	-0.10	**0.13**	-0.09	0.04	0.03	**0.28**	**-0.19**	**0.12**	**0.35**	**0.51**	**0.22**
19. Self-Confidence (FFMI)	**0.56**	**0.31**	**0.44**	**-0.59**	**0.34**	-0.09	**0.21**	-0.05	**0.13**	**0.21**	**0.48**	**-0.19**	**0.60**	**0.61**	-0.03	**0.57**
20. Manipulative (FFMI)	0.03	**-0.41**	**-0.24**	**0.13**	0.04	**0.49**	**-0.37**	**0.41**	**-0.14**	**0.34**	-0.07	**0.40**	0.06	**-0.14**	**-0.27**	**-0.16**
21. Callousness (FFMI)	**-0.12**	**-0.58**	**-0.22**	0.06	**-0.19**	**0.46**	**-0.14**	**0.43**	-0.08	**0.18**	-0.10	**0.58**	0.01	**-0.15**	**-0.19**	-0.07
22. Cynical (FFMI)	**-0.26**	**-0.49**	**-0.18**	**0.29**	**-0.14**	**0.31**	**-0.28**	**0.28**	**-0.52**	0.02	**-0.24**	**0.32**	-0.08	**-0.24**	-0.01	**-0.20**
23. Affective Instability (PAI-BOR)	**-0.29**	**-0.48**	**-0.48**	**0.74**	**-0.12**	**0.27**	**-0.19**	**0.24**	**-0.17**	0.06	**-0.39**	**0.25**	**-0.21**	**-0.46**	**-0.22**	**-0.65**
24. Identity Problems (PAI-BOR)	**-0.26**	**-0.33**	**-0.45**	**0.68**	-0.09	**0.25**	**-0.14**	**0.21**	**-0.14**	**0.14**	**-0.35**	**0.20**	**-0.27**	**-0.50**	**-0.18**	**-0.61**
25. Negative Relationships (PAI-BOR)	**-0.23**	**-0.43**	**-0.37**	**0.59**	-0.03	**0.29**	**-0.16**	**0.28**	**-0.21**	**0.15**	**-0.29**	**0.20**	**-0.17**	**-0.39**	**-0.22**	**-0.51**
26. Self-Harm (PAI-BOR)	-0.03	**-0.35**	**-0.53**	**0.34**	-0.07	**0.22**	**-0.13**	**0.25**	-0.08	0.09	**-0.25**	**0.26**	-0.09	**-0.33**	**-0.49**	**-0.38**
27. Anxious Uncertainty (FFBI)	**-0.27**	**-0.28**	**-0.37**	**0.81**	-0.09	**0.16**	**-0.16**	**0.11**	**-0.13**	0.06	**-0.30**	0.09	**-0.31**	**-0.45**	-0.07	**-0.67**
28. Dysregulated Anger (FFBI)	**-0.13**	**-0.47**	**-0.43**	**0.59**	-0.08	**0.27**	**-0.15**	**0.25**	**-0.14**	**0.12**	**-0.27**	**0.23**	-0.10	**-0.35**	**-0.25**	**-0.52**
29. Despondence (FFBI)	**-0.30**	**-0.33**	**-0.45**	**0.71**	-0.05	**0.15**	**-0.19**	0.10	**-0.14**	-0.01	**-0.43**	**0.14**	**-0.27**	**-0.51**	**-0.13**	**-0.61**
30. Self-Disturbance (FFBI)	**-0.34**	**-0.47**	**-0.46**	**0.65**	-0.08	**0.31**	**-0.21**	**0.26**	**-0.16**	0.06	**-0.42**	**0.27**	**-0.28**	**-0.55**	**-0.15**	**-0.58**
31. Behavioral Dysregulation (FFBI)	**-0.14**	**-0.46**	**-0.52**	**0.50**	-0.05	**0.34**	**-0.17**	**0.32**	**-0.11**	0.10	**-0.31**	**0.33**	**-0.14**	**-0.43**	**-0.42**	**-0.50**
32. Affective Dysregulation (FFBI)	**-0.20**	**-0.43**	**-0.48**	**0.76**	**-0.11**	**0.27**	**-0.18**	**0.24**	**-0.14**	0.08	**-0.35**	**0.24**	**-0.20**	**-0.46**	**-0.27**	**-0.70**
33. Fragility (FFBI)	**-0.21**	**-0.40**	**-0.46**	**0.62**	-0.10	**0.29**	**-0.18**	**0.26**	**-0.13**	0.09	**-0.36**	**0.27**	**-0.20**	**-0.45**	**-0.21**	**-0.58**
34. Dissociative Tendencies (FFBI)	**-0.21**	**-0.37**	**-0.44**	**0.55**	-0.06	**0.26**	**-0.16**	**0.24**	**-0.12**	**0.11**	**-0.32**	**0.25**	**-0.22**	**-0.44**	**-0.18**	**-0.52**
35. Distrustfulness (FFBI)	**-0.28**	**-0.50**	**-0.31**	**0.57**	-0.06	**0.35**	**-0.19**	**0.30**	**-0.31**	0.09	**-0.28**	**0.24**	**-0.18**	**-0.37**	**-0.13**	**-0.44**
36. Manipulativeness (FFBI)	-0.08	**-0.56**	**-0.40**	**0.31**	-0.10	**0.42**	**-0.33**	**0.40**	**-0.15**	**0.20**	**-0.21**	**0.46**	-0.04	**-0.27**	**-0.31**	**-0.29**
37. Oppositional (FFBI)	-0.09	**-0.63**	**-0.42**	**0.43**	**-0.13**	**0.33**	**-0.21**	**0.32**	**-0.17**	**0.14**	**-0.24**	**0.43**	-0.02	**-0.34**	**-0.29**	**-0.40**
38. Rashness (FFBI)	-0.05	**-0.43**	**-0.51**	**0.39**	**-0.11**	**0.28**	**-0.13**	**0.31**	-0.06	0.08	**-0.28**	**0.33**	-0.10	**-0.35**	**-0.55**	**-0.40**

Table S2 (continued).

Correlations between Machiavellianism, Borderline Personality, and the Big Five

	17	18	19	20	21	22	23	24	25	26	27	28	29	30	31	32	33	34	35	36	37
18. Order (FFMI)	-0.06	—																			
19. Self-Confidence (FFMI)	**0.25**	**0.15**	—																		
20. Manipulative (FFMI)	**0.46**	**-0.16**	0.01	—																	
21. Callousness (FFMI)	**0.43**	**-0.17**	-0.02	**0.43**	—																
22. Cynical (FFMI)	**0.16**	-0.04	**-0.23**	**0.29**	**0.26**	—															
23. Affective Instability (PAI-BOR)	0.08	**-0.23**	**-0.45**	**0.24**	**0.20**	**0.31**	—														
24. Identity Problems (PAI-BOR)	0.05	**-0.22**	**-0.45**	**0.23**	**0.13**	**0.24**	**0.72**	—													
25. Negative Relationships (PAI-BOR)	**0.11**	**-0.22**	**-0.34**	**0.28**	**0.19**	**0.37**	**0.66**	**0.70**	—												
26. Self-Harm (PAI-BOR)	**0.14**	**-0.43**	**-0.19**	**0.27**	**0.21**	**0.14**	**0.52**	**0.49**	**0.42**	—											
27. Anxious Uncertainty (FFBI)	-0.04	-0.10	**-0.54**	**0.14**	-0.02	**0.23**	**0.61**	**0.70**	**0.54**	**0.30**	—										
28. Dysregulated Anger (FFBI)	0.10	**-0.24**	**-0.29**	**0.27**	**0.24**	**0.25**	**0.73**	**0.53**	**0.53**	**0.50**	**0.50**	—									
29. Despondence (FFBI)	-0.01	**-0.22**	**-0.51**	**0.14**	0.05	**0.22**	**0.70**	**0.72**	**0.61**	**0.42**	**0.68**	**0.53**	—								
30. Self-Disturbance (FFBI)	0.09	**-0.21**	**-0.50**	**0.29**	**0.23**	**0.30**	**0.68**	**0.71**	**0.66**	**0.43**	**0.63**	**0.59**	**0.73**	—							
31. Behavioral Dysregulation (FFBI)	**0.13**	**-0.33**	**-0.28**	**0.33**	**0.29**	**0.21**	**0.64**	**0.59**	**0.56**	**0.65**	**0.49**	**0.70**	**0.56**	**0.63**	—						
32. Affective Dysregulation (FFBI)	0.08	**-0.25**	**-0.43**	**0.25**	**0.20**	**0.26**	**0.83**	**0.72**	**0.61**	**0.52**	**0.69**	**0.74**	**0.72**	**0.72**	**0.72**	—					
33. Fragility (FFBI)	**0.11**	**-0.24**	**-0.39**	**0.23**	**0.21**	**0.24**	**0.75**	**0.70**	**0.63**	**0.54**	**0.58**	**0.62**	**0.78**	**0.69**	**0.65**	**0.76**	—				
34. Dissociative Tendencies (FFBI)	**0.11**	**-0.24**	**-0.35**	**0.23**	**0.22**	**0.22**	**0.67**	**0.67**	**0.56**	**0.48**	**0.55**	**0.55**	**0.70**	**0.71**	**0.60**	**0.70**	**0.75**	—			
35. Distrustfulness (FFBI)	**0.13**	**-0.14**	**-0.34**	**0.30**	**0.27**	**0.50**	**0.59**	**0.62**	**0.71**	**0.34**	**0.57**	**0.53**	**0.58**	**0.67**	**0.58**	**0.61**	**0.60**	**0.58**	—		
36. Manipulativeness (FFBI)	**0.33**	**-0.25**	**-0.14**	**0.55**	**0.47**	**0.25**	**0.47**	**0.39**	**0.45**	**0.47**	**0.29**	**0.52**	**0.34**	**0.49**	**0.65**	**0.51**	**0.52**	**0.48**	**0.50**	—	
37. Oppositional (FFBI)	**0.22**	**-0.25**	**-0.22**	**0.32**	**0.47**	**0.27**	**0.58**	**0.44**	**0.49**	**0.47**	**0.35**	**0.68**	**0.40**	**0.51**	**0.61**	**0.59**	**0.55**	**0.50**	**0.48**	**0.63**	—
38. Rashness (FFBI)	**0.13**	**-0.40**	**-0.19**	**0.30**	**0.30**	**0.14**	**0.55**	**0.45**	**0.47**	**0.64**	**0.38**	**0.63**	**0.44**	**0.50**	**0.80**	**0.62**	**0.55**	**0.50**	**0.48**	**0.64**	**0.61**

Note. Bold correlations are $p < .001$.

Figure S1.

Elbow plot.

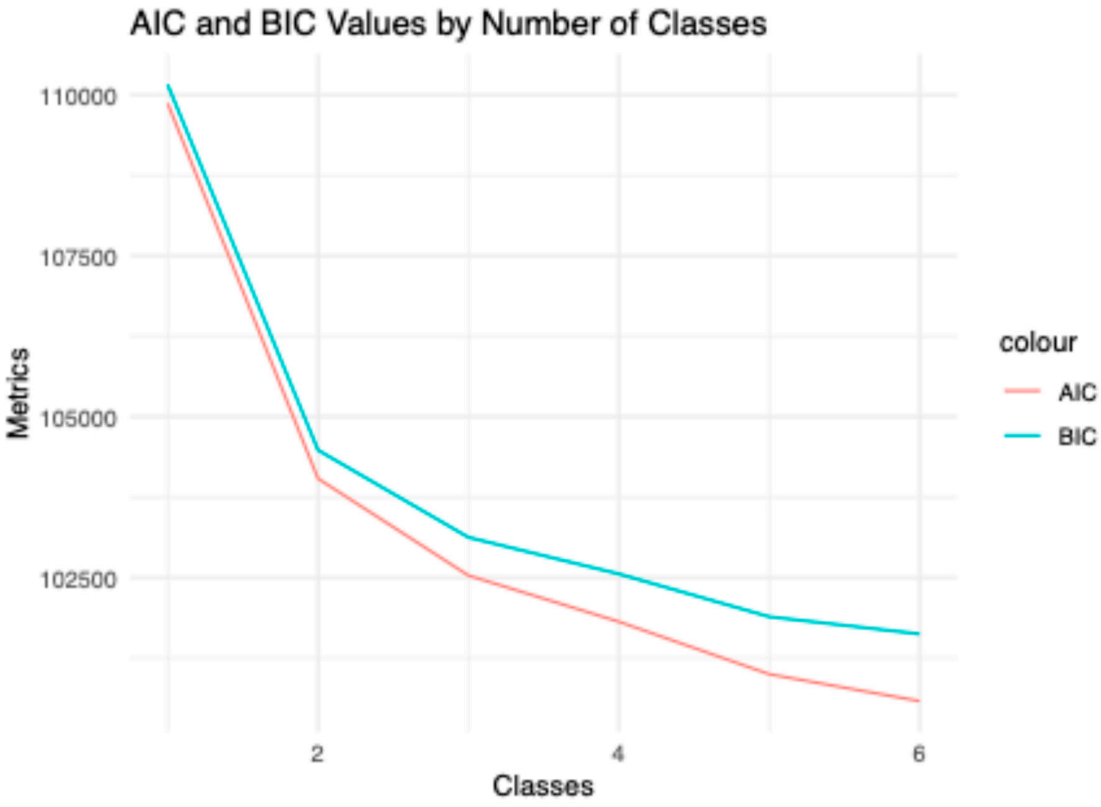

Supplement: Supplementary file 1 [file ejihpe-15-00185-s001.zip › ejihpe-3694201-supplementary.pdf]
